# Supplementary material for: Synthesis and Antiviral Activity of Camphene Derivatives against Different Types of Viruses
Source: Molecules. 2021 Apr 13;26(8):2235. doi: 10.3390/molecules26082235 (PMC8070564; doi:10.3390/molecules26082235)
Supplement: Supplementary file 1 [file molecules-26-02235-s001.pdf]

## Supplementary Information

*Article*

# Synthesis and antiviral activity of camphene derivatives against different types of viruses

**Anastasiya S. Sokolova[1*], Valentina P. Putilova[1], Olga I. Yarovaya[1], Anastasiya V. Zybkina[2], Ekaterina D. Mordvinova[1,2], Anna V. Zaykovskaya[2], Dmitriy N. Shcherbakov[2], Iana R. Orshanskaya[3], Ekaterina O. Sinegubova[3], Iana L. Esaulkova[3], Sophia S. Borisevich[4], Nikolay I. Bormotov[2], Larisa N. Shishkina[2], Vladimir V. Zarubaev[3], Oleg V. Pyankov[2], Rinat A. Maksyutov[2], Nariman F. Salakhutdinov[1]**

1 N.N. Vorozhtsov Novosibirsk Institute of Organic chemistry SB RAS, 630090, Russia, Novosibirsk, Lavrent'ev av., 9
2 State Research Center of Virology and Biotechnology VECTOR, Rospotrebnadzor, 630559, Russia, Koltsovo, Novosibirsk Region
3 Pasteur Institute of Epidemiology and Microbiology, 14 Mira str., 197101 St. Petersburg, Russia
4 Laboratory of Chemical Physics, Ufa Institute of Chemistry Ufa Federal Research Center, 71 Pr. Oktyabrya, 450078, Ufa, Russia
* Correspondence: Dr. Anastasiya S. Sokolova, PhD, Tel.: +7-383-330-88-70, e-mail: asokolova@nioch.nsc.ru

## Contents

**Pharmacophore model**........................................2

**NMR $^{1}$H and $^{13}$C spectra**........................................4

## Pharmacophore model

To create a pharmacophore model, we used the Pharmacophore Modeling module implemented in the Schrodinger Suite: Release 2020-4 software. As a result, two models were developed: (i) pharmacophore model based on assessing the contributions of amino acid residues located at M090-site, TBQH-site, and Toremifene-site; (ii) pharmacophore model based on assessing the complementarity of a ligand and a protein at a potential binding site. Also, the pharmacophore profile of the compounds **2a** and **7a** was evaluated (**Figure S1**).

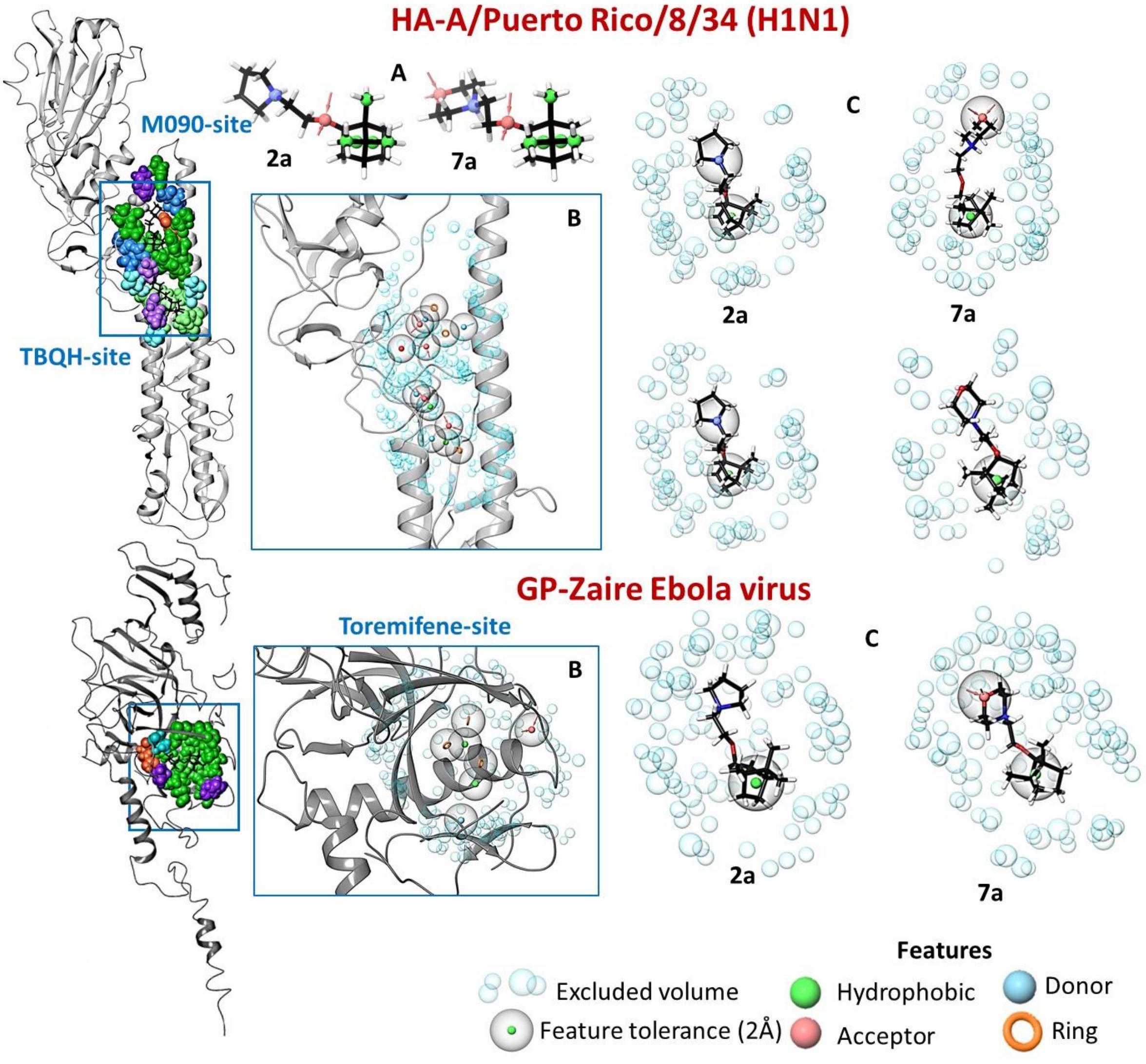


**Figure S1.** (**A**): the pharmacophore profile of the compounds **2a** and **7a**; (**B**): the pharmacophore model based on assessing the contributions of amino acid residues located at M090-site, TBQH-site, and Toremifene-site; **C** the pharmacophore model based on assessing the complementarity of a ligand and a protein.

Compounds **2a** and **7a** have a similar pharmacophore profile and include hydrophobic 1,7,7-trimethylbicyclo[2.2.1]heptan fragment, hydrogen bond acceptor (ether group), and

protonated nitrogen atoms that create an additional reaction center of the molecule capable of forming salt bridges and cation-π stacking interactions.

A pharmacophore model of potential ligand-binding sites shows that surrounding amino acids have hydrophobic characteristics (shown in green in **Figure 1S-B**) and there are a number of amino acids that may contain atoms or groups of atoms exhibiting the properties of acceptors or donors of hydrogen bonds, as well as aromatic rings. The location of such features as hydrogen bond acceptors or donors, hydrophobic, and aromatic groups in the binding sites of influenza and Ebola viruses differ in space. However, saturation with hydrophobic amino acids increases the likelihood that compounds containing a hydrophobic moiety, such as 1,7,7-trimethylbicyclo[2.2.1]heptan moiety in compounds **2a** and **7a**, will bind. The presence of acceptor and donor features allows hydrogen and salt bridges to be formed, which are observed when compounds **2a** and **7a** bind at the described binding sites. In addition, aromatic amino acids can form stacking interactions with protonated pyrolidine and/or morpholic fragments of the compounds **2a** and **7a**.

The pharmacophore model based on the principle of ligand-protein complementarity (**Figure 1S-C**) in all cases contains a hydrophobic feature: ligands bind to the binding sites by forming strong hydrophobic contacts with amino acids with similar features being formed.

# NMR $^{1}H$ and $^{13}C$ spectra

## Compound 2a

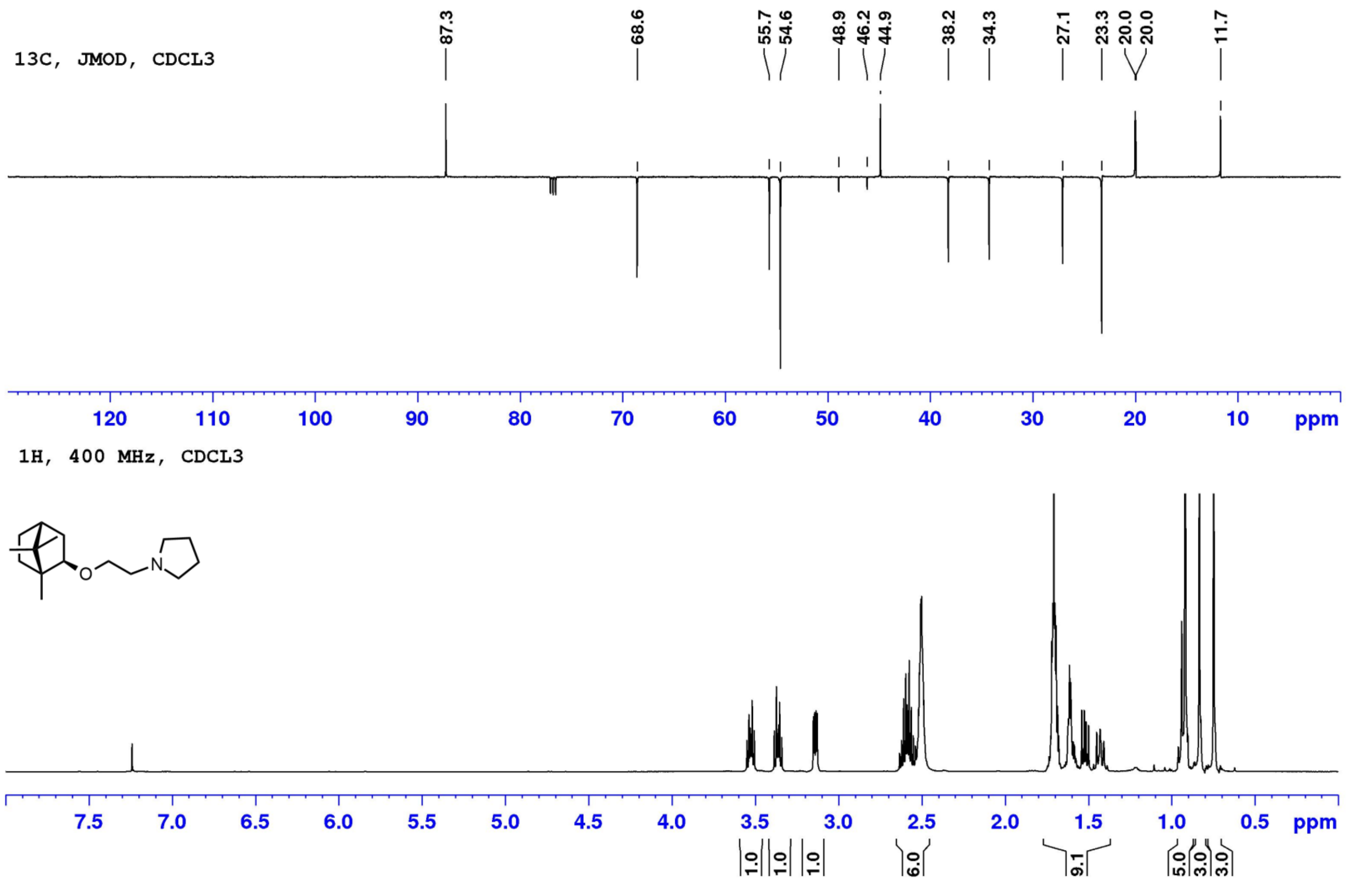

**Compound 2b**

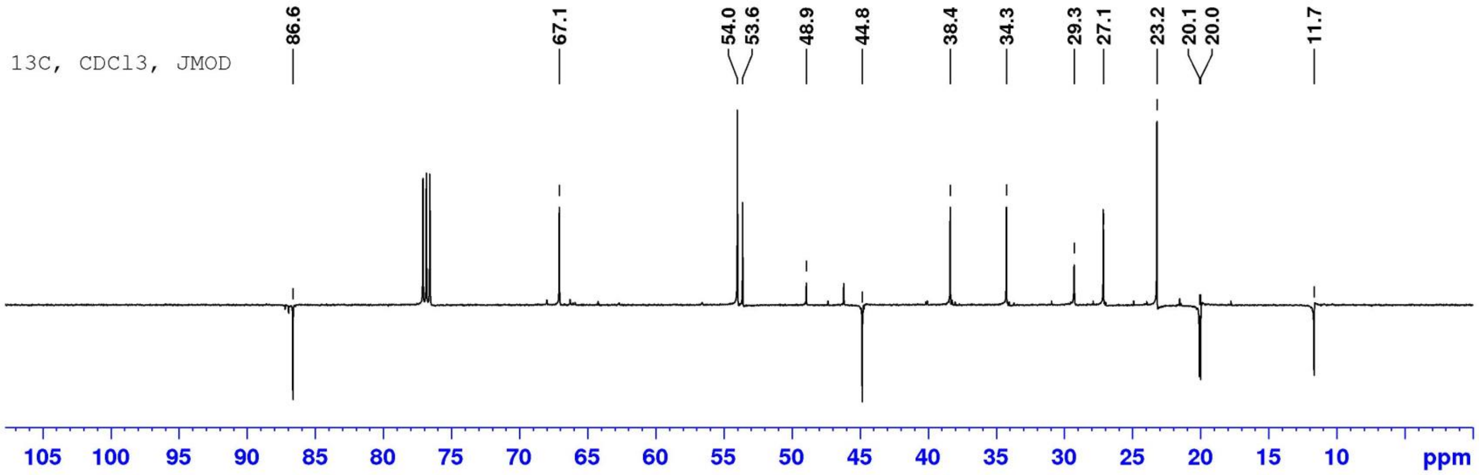
13C, CDCl3, JMOD
86.6
67.1
54.0
53.6
48.9
44.8
38.4
34.3
29.3
27.1
23.2
20.1
20.0
11.7
105 100 95 90 85 80 75 70 65 60 55 50 45 40 35 30 25 20 15 10 ppm

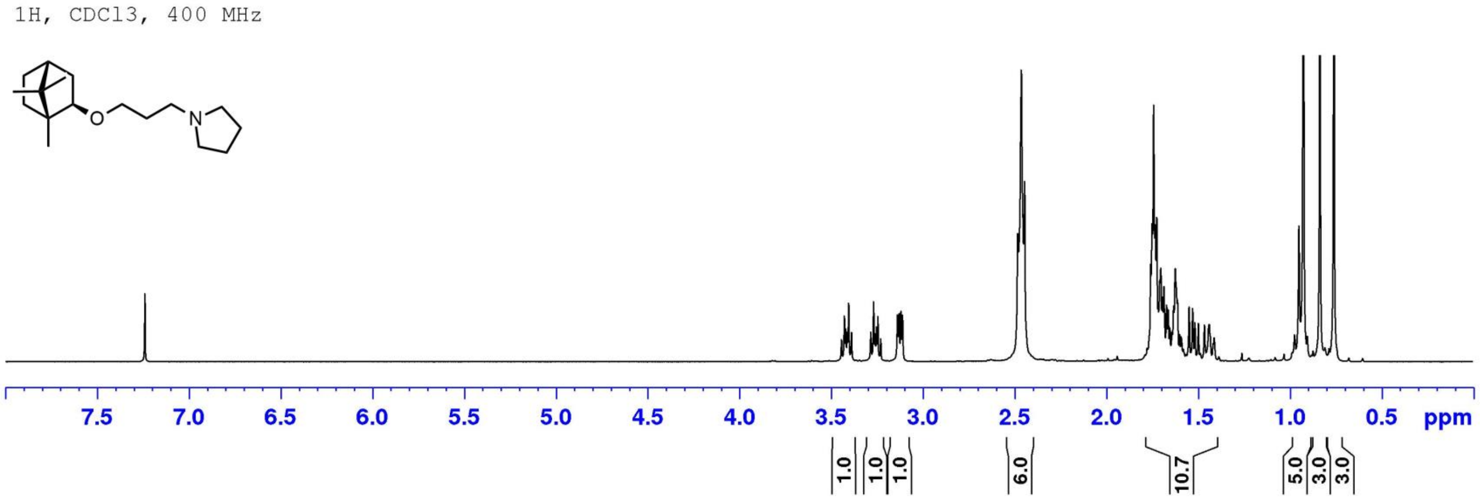
1H, CDCl3, 400 MHz
O
N
7.5 7.0 6.5 6.0 5.5 5.0 4.5 4.0 3.5 3.0 2.5 2.0 1.5 1.0 0.5 ppm
1.0
1.0
1.0
6.0
10.7
5.0
3.0
3.0

**Compound 3a**

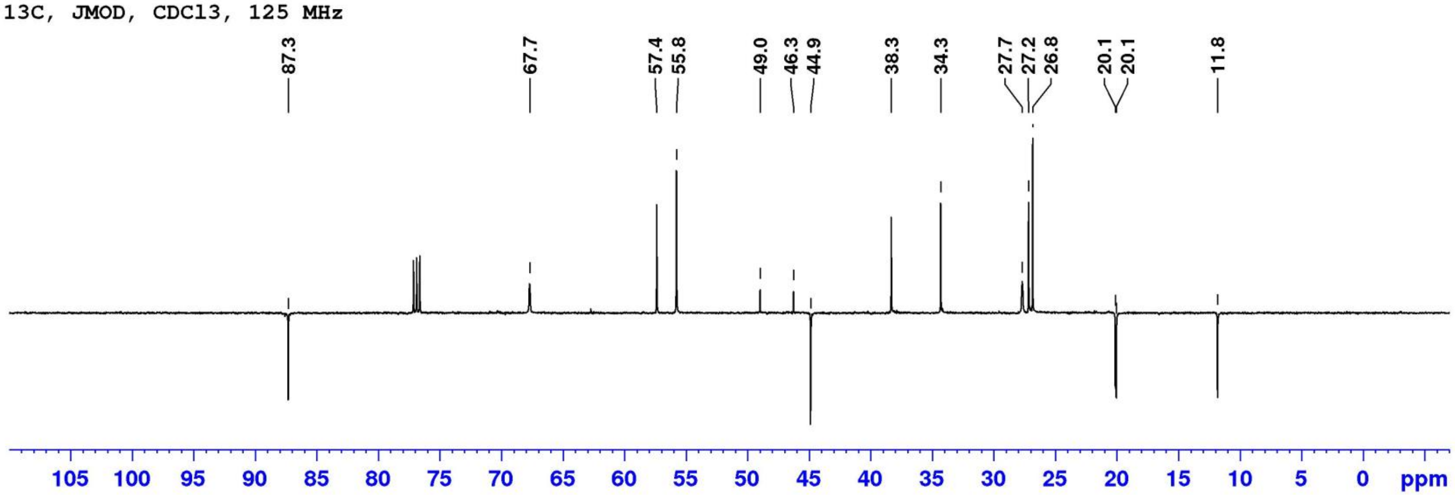
13C, JMOD, CDCl3, 125 MHz
87.3
67.7
57.4
55.8
49.0
46.3
44.9
38.3
34.3
27.7
27.2
26.8
20.1
20.1
11.8
105 100 95 90 85 80 75 70 65 60 55 50 45 40 35 30 25 20 15 10 5 0 ppm

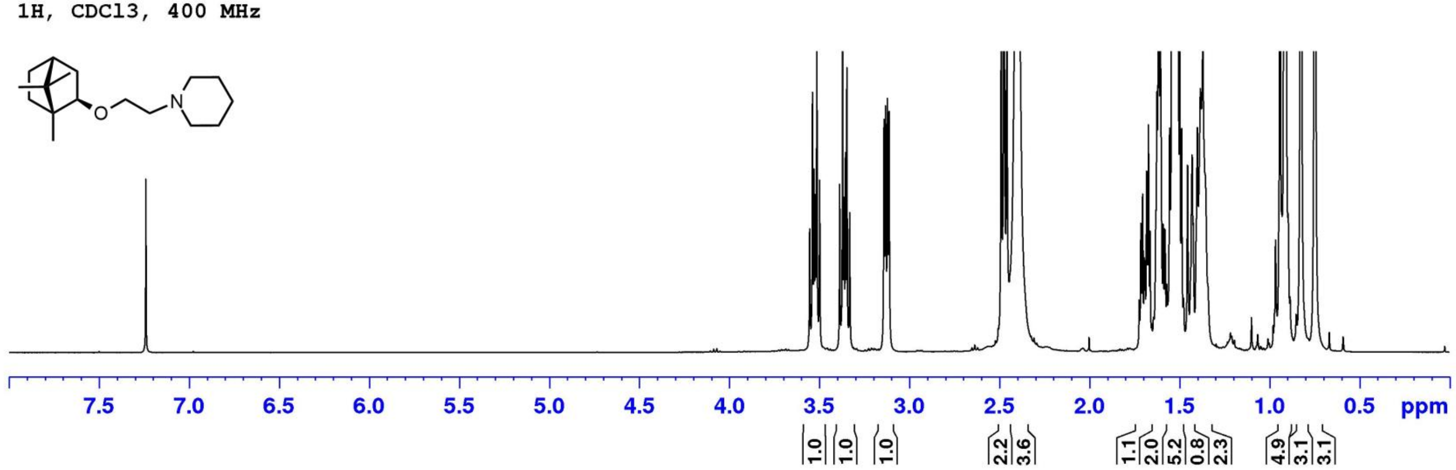
1H, CDCl3, 400 MHz
O
N
7.5 7.0 6.5 6.0 5.5 5.0 4.5 4.0 3.5 3.0 2.5 2.0 1.5 1.0 0.5 ppm
1.0
1.0
1.0
2.2
3.6
1.1
2.0
5.2
0.8
2.3
4.9
3.1
3.1

**Compound 3b**

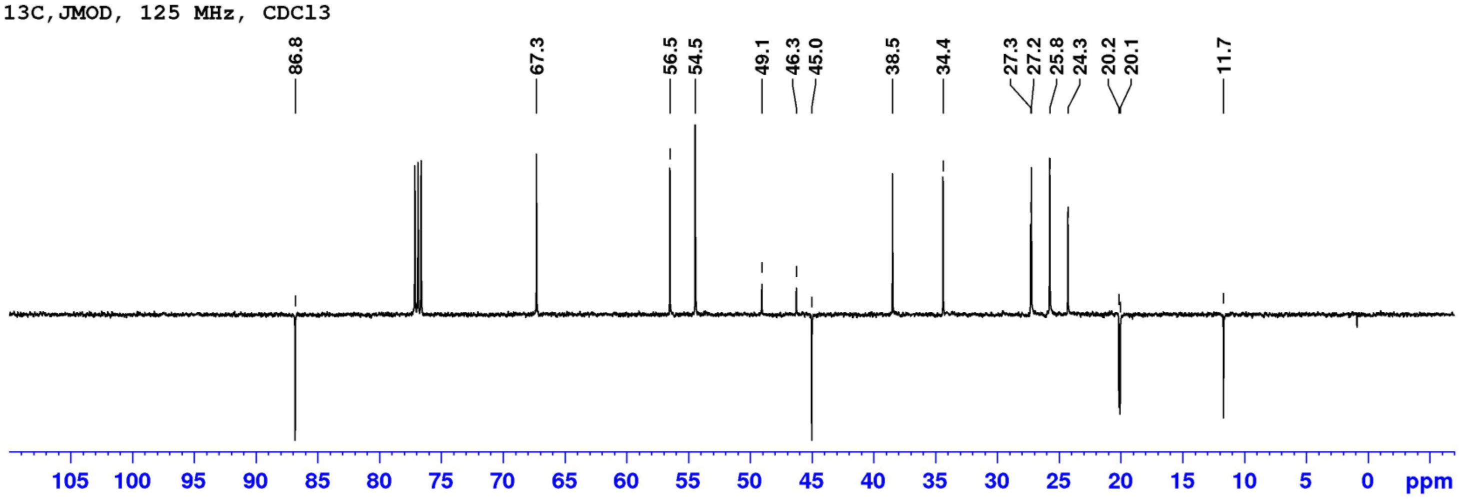
13C,JMOD, 125 MHz, CDCl3
86.8
67.3
56.5
54.5
49.1
46.3
45.0
38.5
34.4
27.3
27.2
25.8
24.3
20.2
20.1
11.7
105 100 95 90 85 80 75 70 65 60 55 50 45 40 35 30 25 20 15 10 5 0 ppm

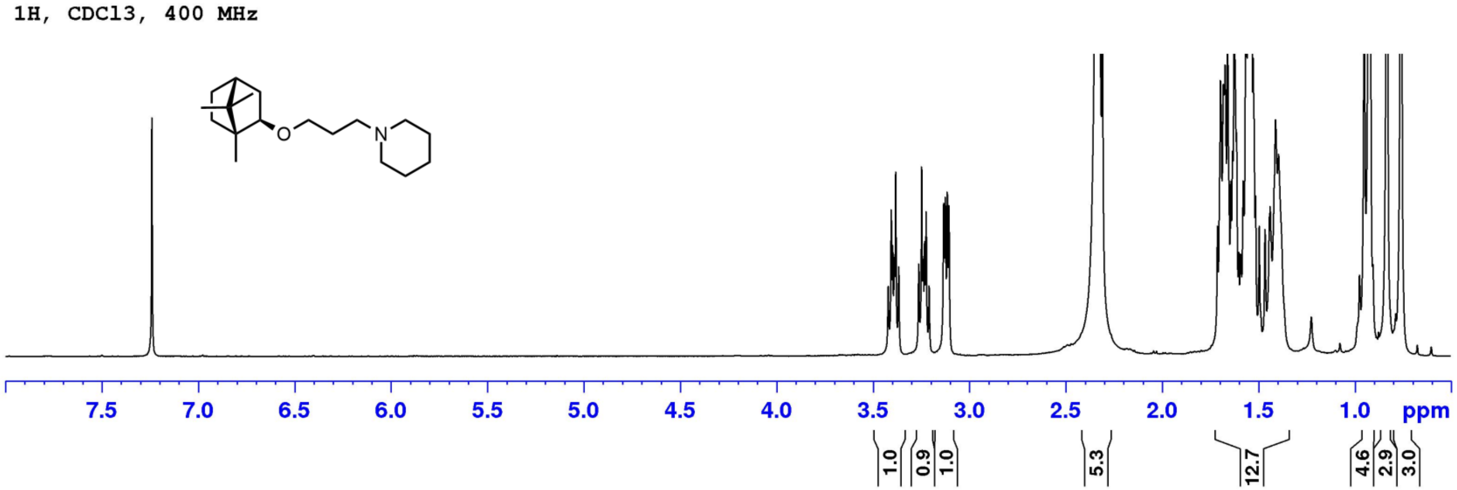
1H, CDCl3, 400 MHz
O
N
7.5 7.0 6.5 6.0 5.5 5.0 4.5 4.0 3.5 3.0 2.5 2.0 1.5 1.0 ppm
1.0
0.9
1.0
5.3
12.7
4.6
2.9
3.0

## Compound 4a

13C, JMOD, 125 MHz, CDCl3

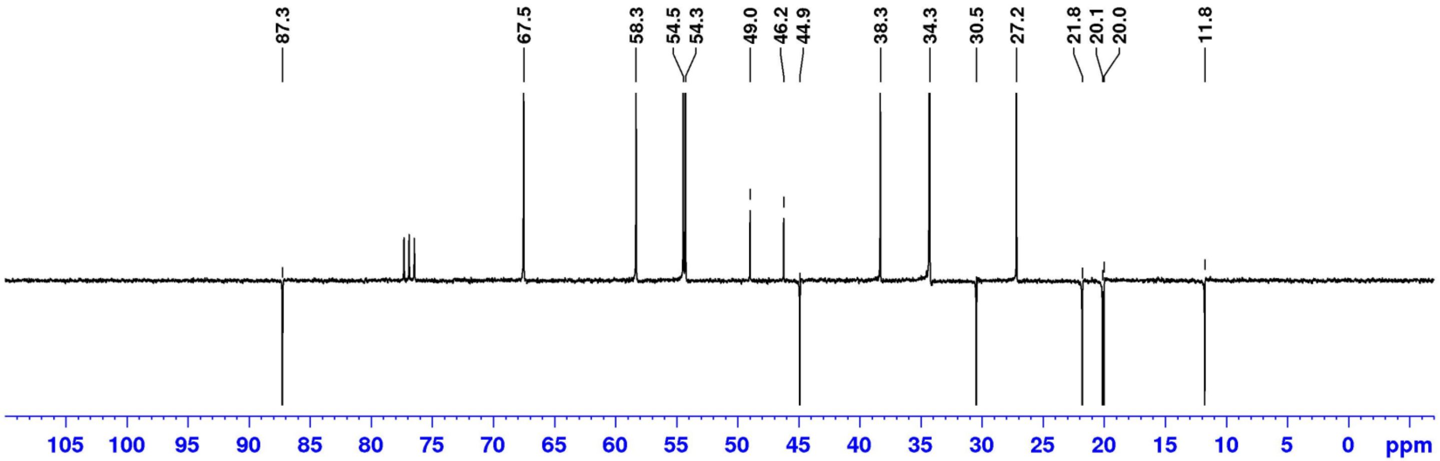


1H, CDCl3, 400 MHz

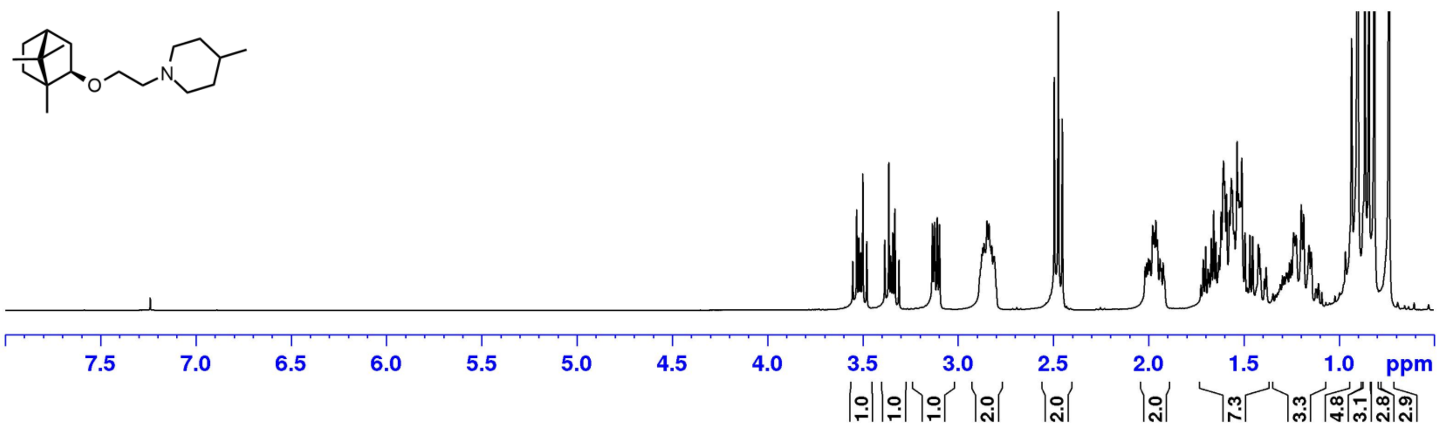

**Compound 4b**

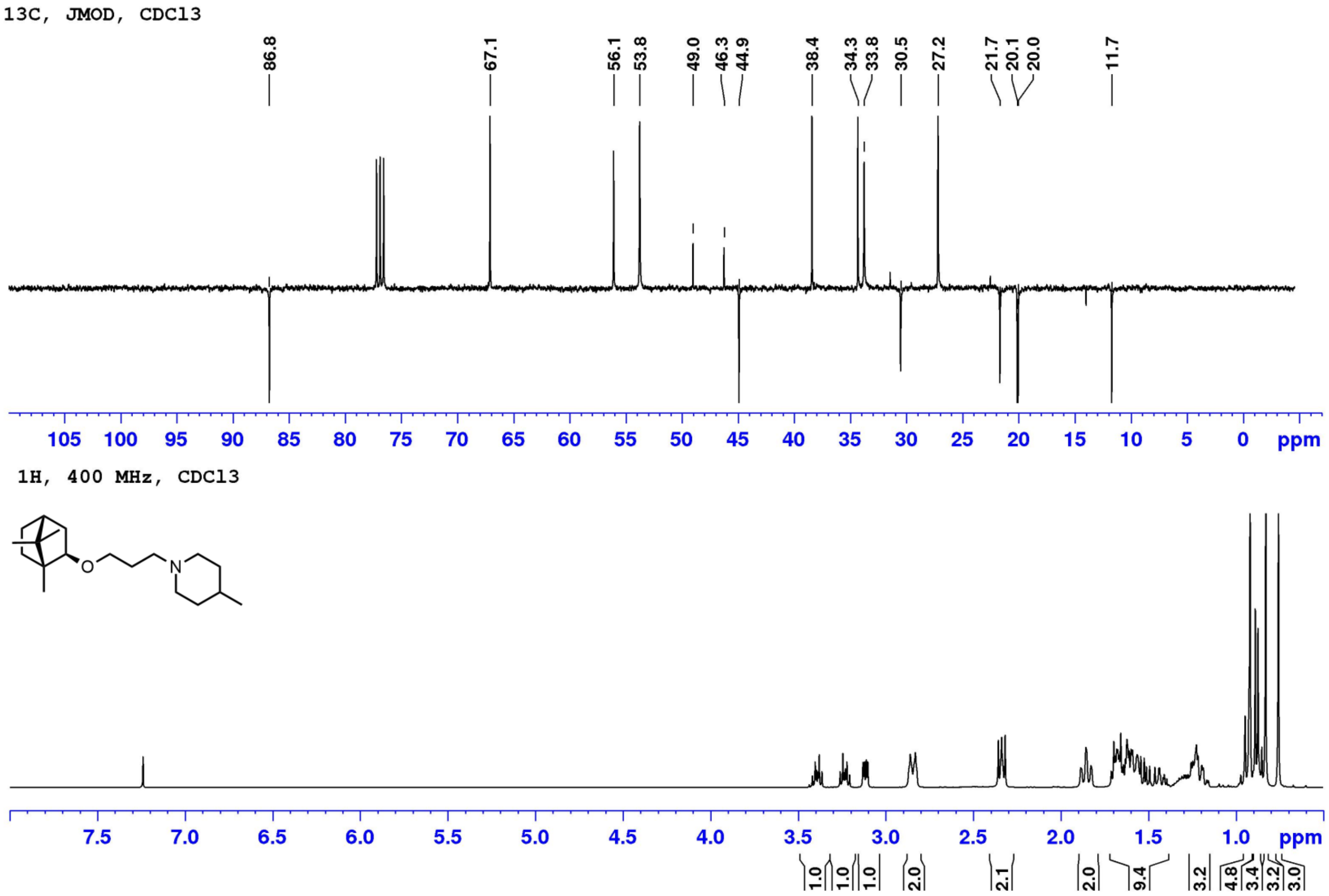
13C, JMOD, CDCl3
86.8
67.1
56.1
53.8
49.0
46.3
44.9
38.4
34.3
33.8
30.5
27.2
21.7
20.1
20.0
11.7
105 100 95 90 85 80 75 70 65 60 55 50 45 40 35 30 25 20 15 10 5 0 ppm
1H, 400 MHz, CDCl3
7.5 7.0 6.5 6.0 5.5 5.0 4.5 4.0 3.5 3.0 2.5 2.0 1.5 1.0 ppm
1.0
1.0
1.0
2.0
2.1
2.0
9.4
3.2
4.8
3.4
3.2
3.0
O
N

**Compound 5a**

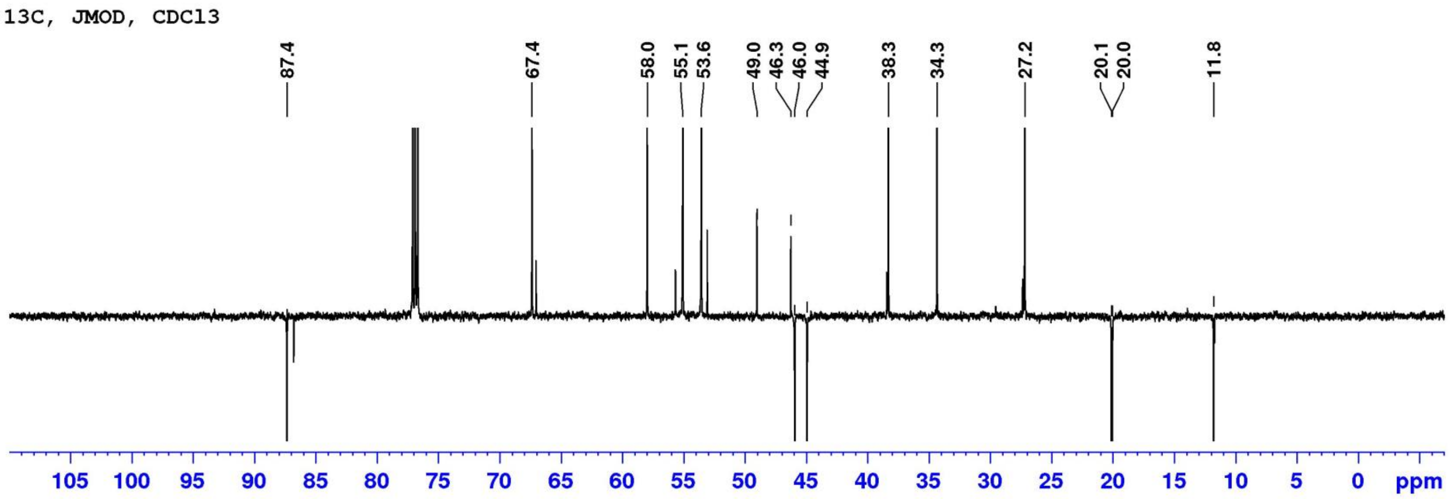

13C, JMOD, CDCl3
87.4
67.4
58.0
55.1
53.6
49.0
46.3
46.0
44.9
38.3
34.3
27.2
20.1
20.0
11.8
105 100 95 90 85 80 75 70 65 60 55 50 45 40 35 30 25 20 15 10 5 0 ppm


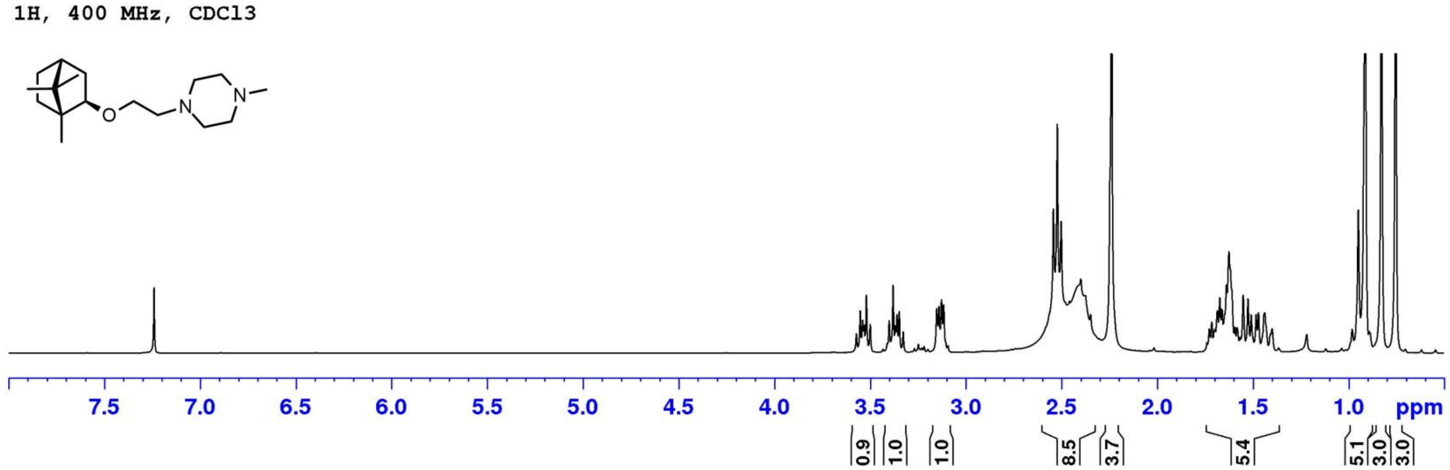

1H, 400 MHz, CDCl3
O
N
N
7.5 7.0 6.5 6.0 5.5 5.0 4.5 4.0 3.5 3.0 2.5 2.0 1.5 1.0 ppm
0.9
1.0
1.0
8.5
3.7
5.4
5.1
3.0
3.0

**Compound 5b**

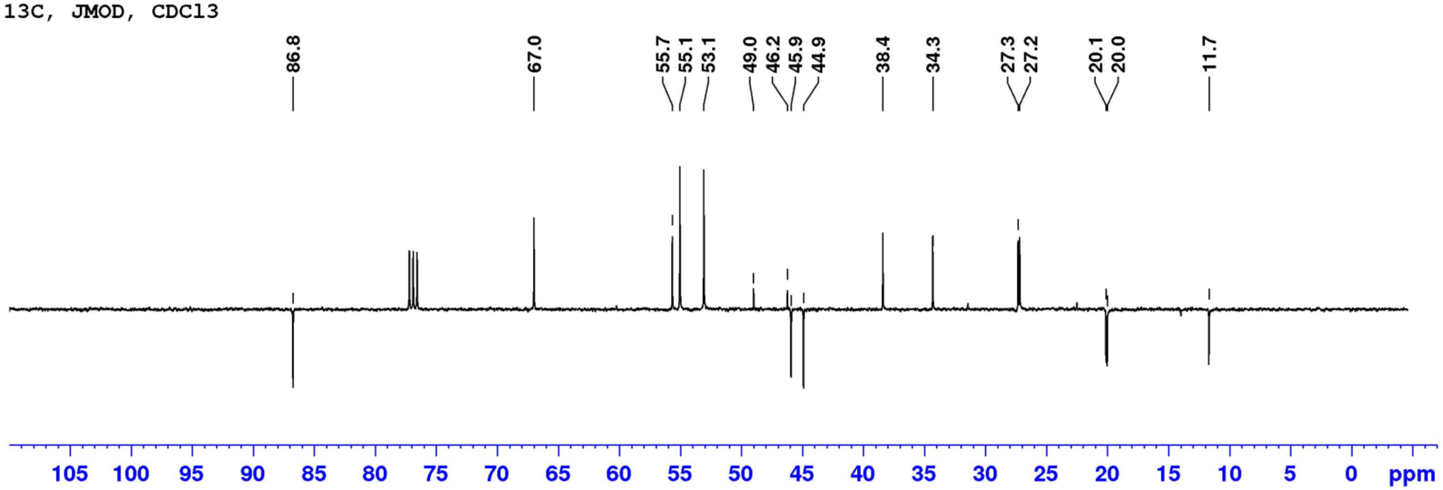
13C, JMOD, CDCl3
86.8
67.0
55.7
55.1
53.1
49.0
46.2
45.9
44.9
38.4
34.3
27.3
27.2
20.1
20.0
11.7
105 100 95 90 85 80 75 70 65 60 55 50 45 40 35 30 25 20 15 10 5 0 ppm

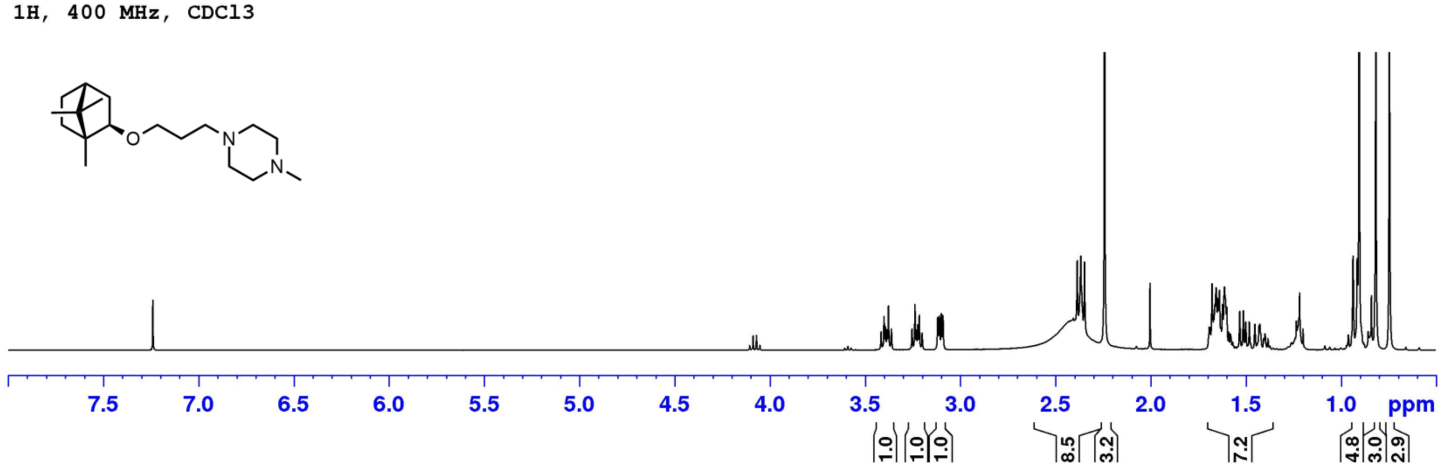
1H, 400 MHz, CDCl3
O
N
N
7.5 7.0 6.5 6.0 5.5 5.0 4.5 4.0 3.5 3.0 2.5 2.0 1.5 1.0 ppm
1.0
1.0
1.0
8.5
3.2
7.2
4.8
3.0
2.9

**Compound 6a**

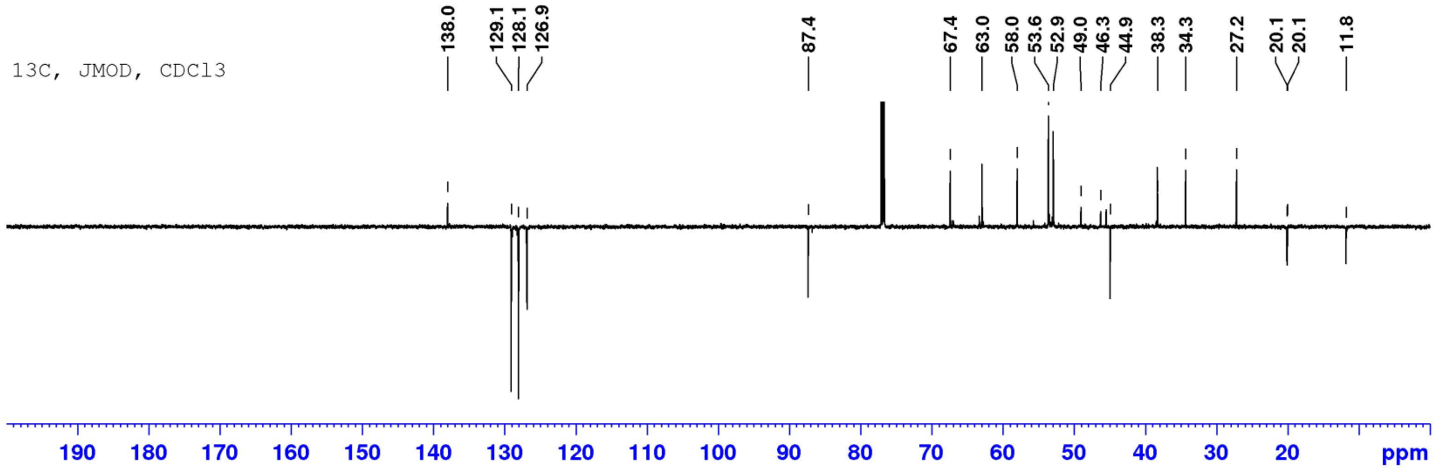
13C, JMOD, CDCl3
138.0
129.1
128.1
126.9
87.4
67.4
63.0
58.0
53.6
52.9
49.0
46.3
44.9
38.3
34.3
27.2
20.1
20.1
11.8
190 180 170 160 150 140 130 120 110 100 90 80 70 60 50 40 30 20 ppm

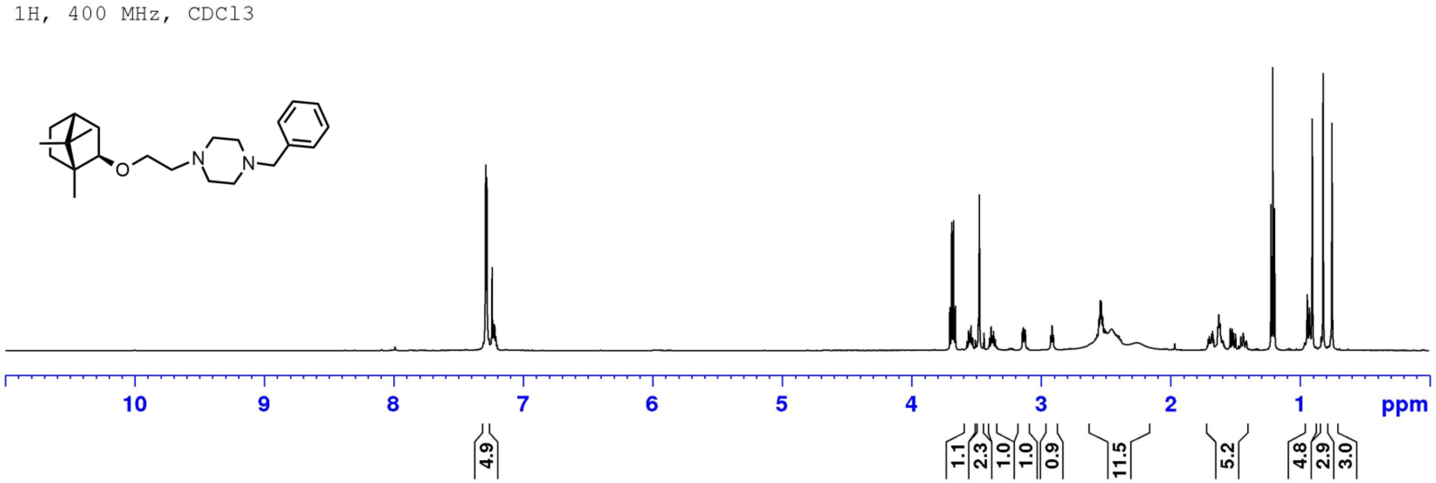
1H, 400 MHz, CDCl3
O
N
N
10 9 8 7 6 5 4 3 2 1 ppm
4.9
1.1
2.3
1.0
1.0
0.9
11.5
5.2
4.8
2.9
3.0

**Compound 6b**

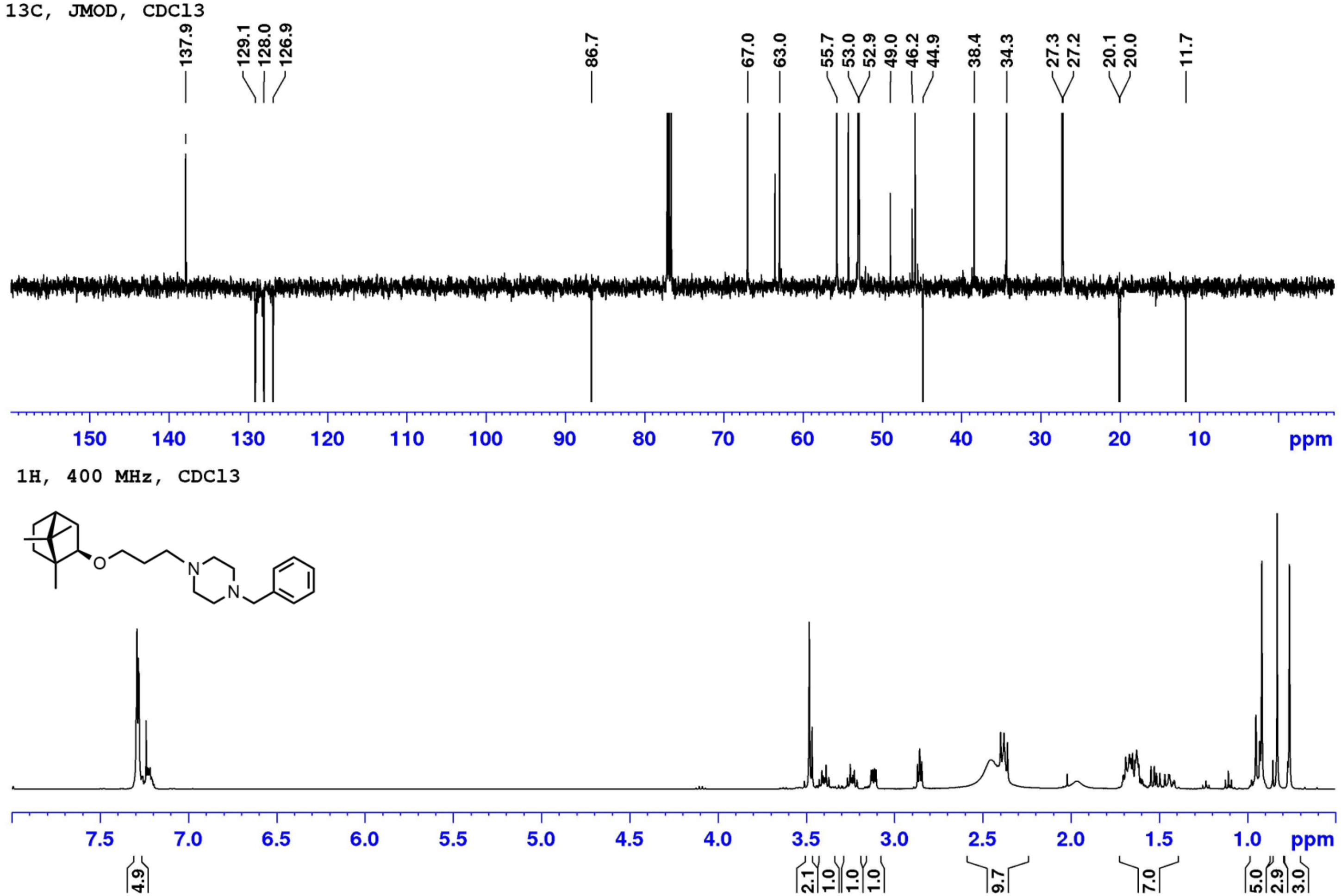
13C, JMOD, CDCl3
137.9
129.1
128.0
126.9
86.7
67.0
63.0
55.7
53.0
52.9
49.0
46.2
44.9
38.4
34.3
27.3
27.2
20.1
20.0
11.7
150 140 130 120 110 100 90 80 70 60 50 40 30 20 10 ppm
1H, 400 MHz, CDCl3
O
N
N
7.5 7.0 6.5 6.0 5.5 5.0 4.5 4.0 3.5 3.0 2.5 2.0 1.5 1.0 ppm
4.9
2.1
1.0
1.0
1.0
9.7
7.0
5.0
2.9
3.0

**Compound 7a**

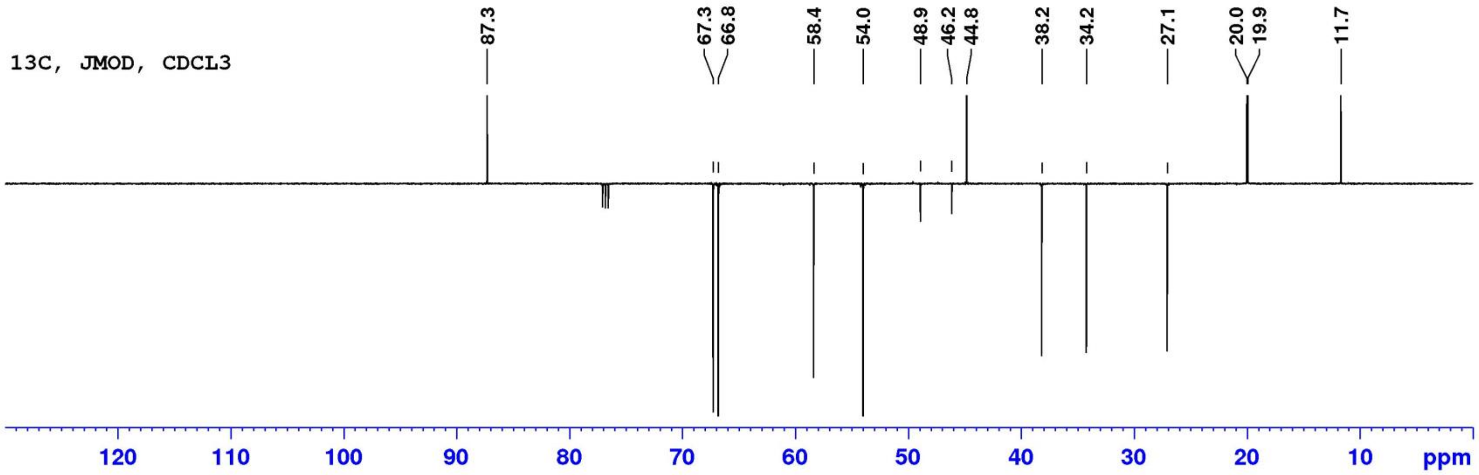
13C, JMOD, CDCL3
87.3
67.3
66.8
58.4
54.0
48.9
46.2
44.8
38.2
34.2
27.1
20.0
19.9
11.7
120 110 100 90 80 70 60 50 40 30 20 10 ppm

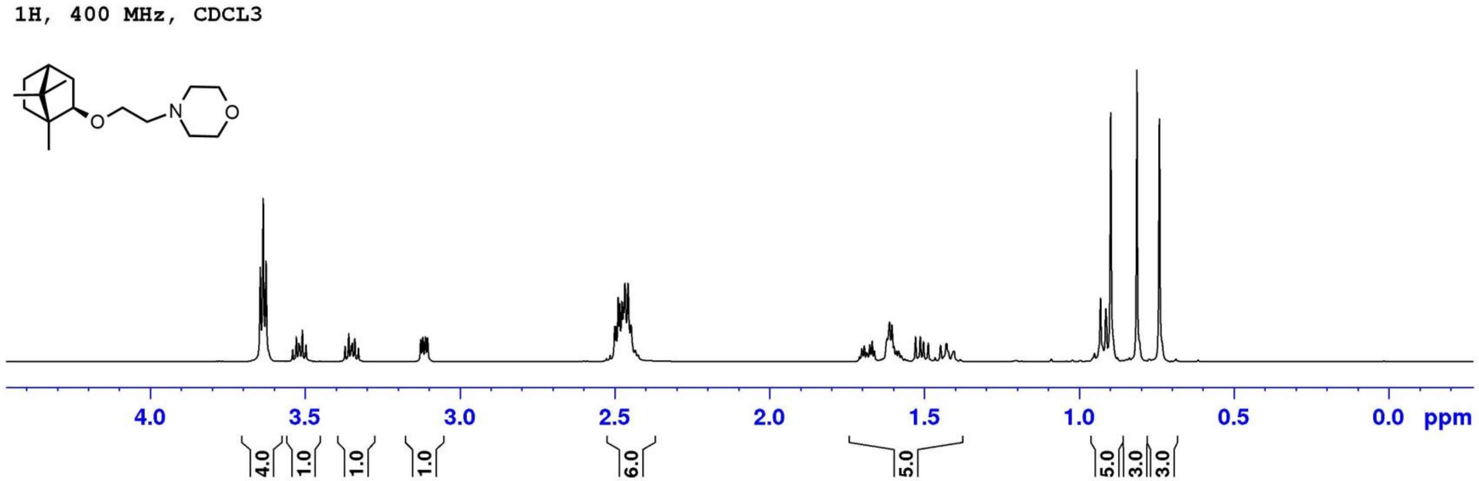
1H, 400 MHz, CDCL3
O
N
O
4.0 3.5 3.0 2.5 2.0 1.5 1.0 0.5 0.0 ppm
4.0
1.0
1.0
1.0
6.0
5.0
5.0
3.0
3.0

**Compound 7b**

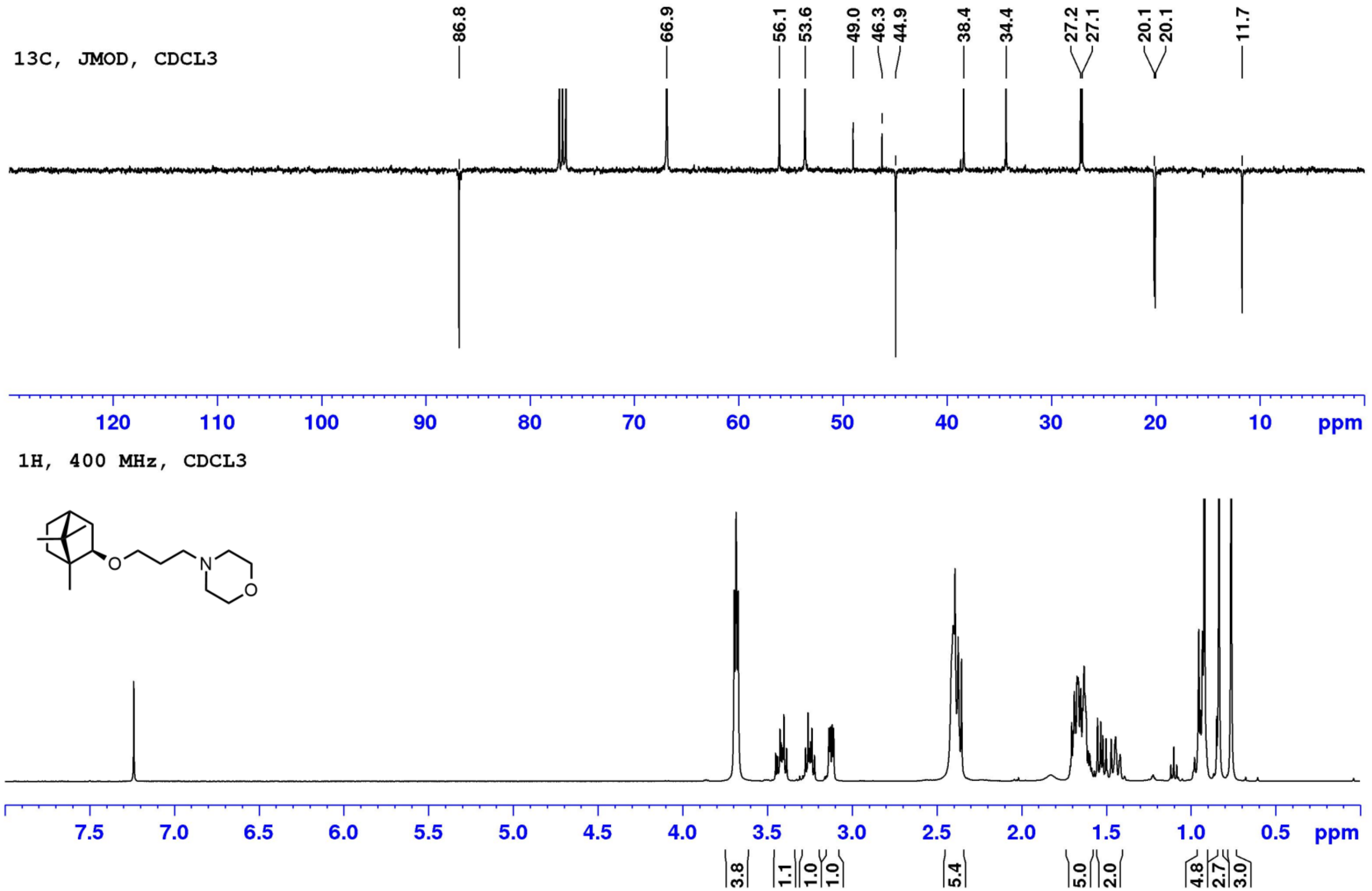
13C, JMOD, CDCL3
86.8
66.9
56.1
53.6
49.0
46.3
44.9
38.4
34.4
27.2
27.1
20.1
20.1
11.7
120 110 100 90 80 70 60 50 40 30 20 10 ppm
1H, 400 MHz, CDCL3
O
N
O
7.5 7.0 6.5 6.0 5.5 5.0 4.5 4.0 3.5 3.0 2.5 2.0 1.5 1.0 0.5 ppm
3.8
1.1
1.0
1.0
5.4
5.0
2.0
4.8
2.7
3.0

**Compound 8a**

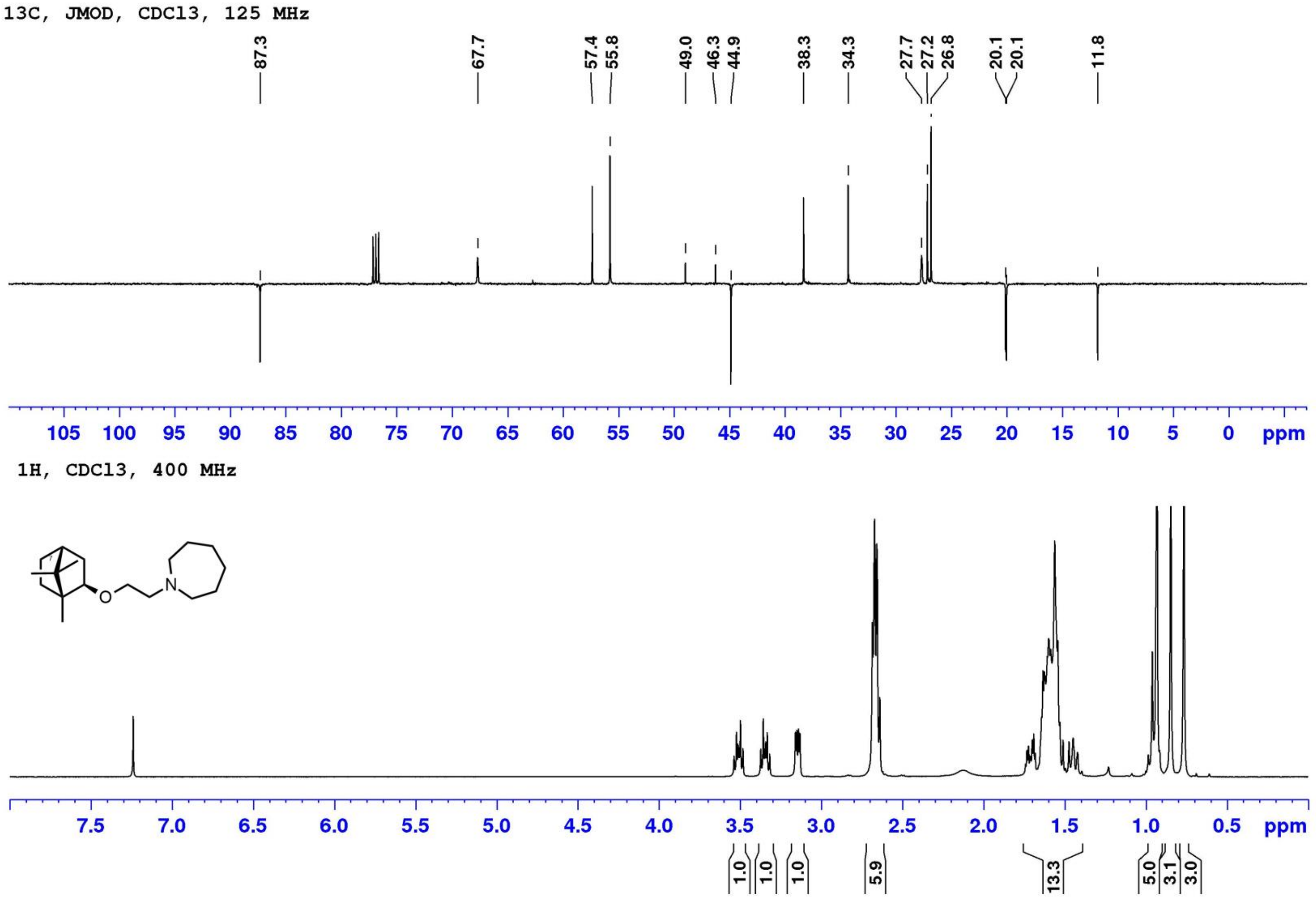
13C, JMOD, CDCl3, 125 MHz
87.3
67.7
57.4
55.8
49.0
46.3
44.9
38.3
34.3
27.7
27.2
26.8
20.1
20.1
11.8
105 100 95 90 85 80 75 70 65 60 55 50 45 40 35 30 25 20 15 10 5 0 ppm
1H, CDCl3, 400 MHz
O
N
7.5 7.0 6.5 6.0 5.5 5.0 4.5 4.0 3.5 3.0 2.5 2.0 1.5 1.0 0.5 ppm
1.0
1.0
1.0
5.9
13.3
5.0
3.1
3.0

**Compound 8b**

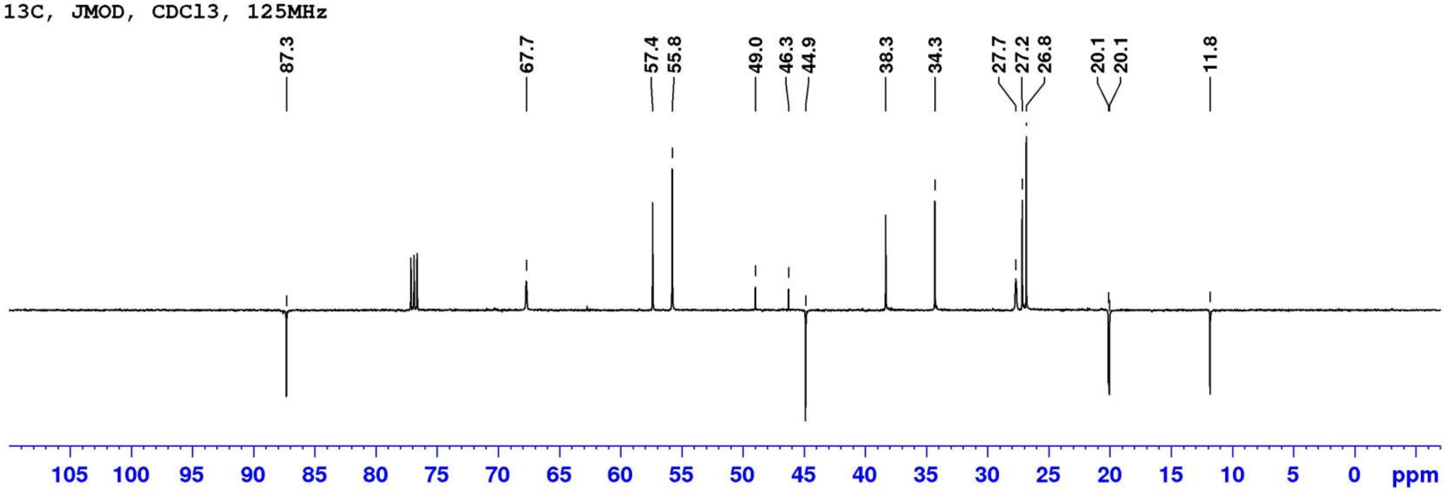
13C, JMOD, CDCl3, 125MHz
87.3
67.7
57.4
55.8
49.0
46.3
44.9
38.3
34.3
27.7
27.2
26.8
20.1
20.1
11.8
105 100 95 90 85 80 75 70 65 60 55 50 45 40 35 30 25 20 15 10 5 0 ppm

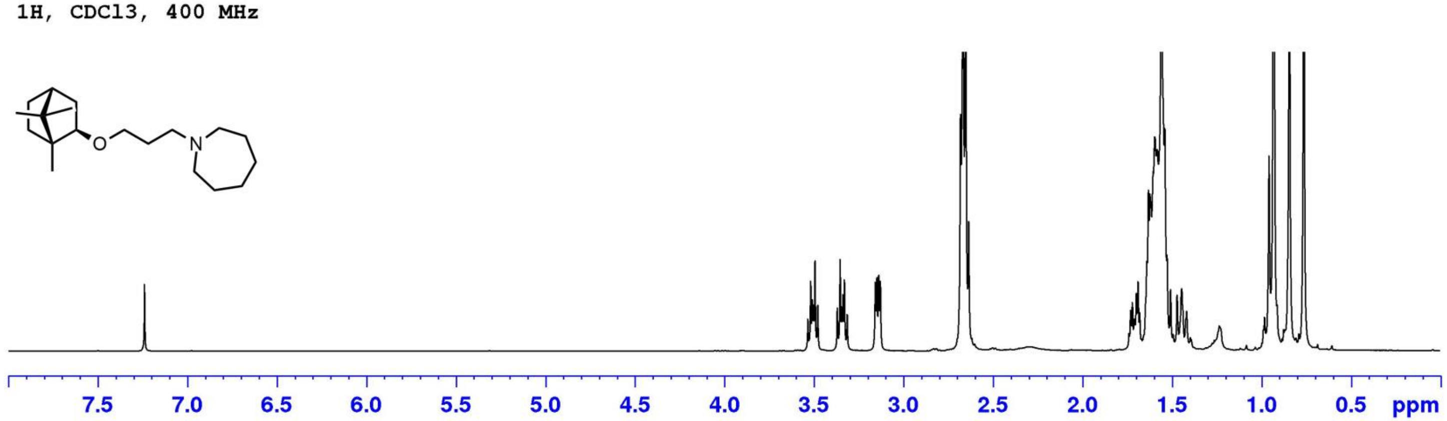
1H, CDCl3, 400 MHz
O
N
7.5 7.0 6.5 6.0 5.5 5.0 4.5 4.0 3.5 3.0 2.5 2.0 1.5 1.0 0.5 ppm
